# Impaired acquisition of novel grapheme-color correspondences in synesthesia

**DOI:** 10.3389/fnhum.2013.00717

**Published:** 2013-10-30

**Authors:** David Brang, Michael Ghiam, Vilayanur S. Ramachandran

**Affiliations:** ^1^Department of Psychology, Northwestern UniversityEvanston, IL, USA; ^2^Department of Psychology, University California San DiegoLa Jolla, CA, USA

**Keywords:** synesthesia, synaesthesia, language, learning, grapheme

## Abstract

Grapheme-color synesthesia is a neurological phenomenon in which letters and numbers (graphemes) consistently evoke particular colors (e.g., A may be experienced as red). These sensations are thought to arise through the cross-activation of grapheme processing regions in the fusiform gyrus and color area V4, supported by anatomical and functional imaging. However, the developmental onset of grapheme-color synesthesia remains elusive as research in this area has largely relied on self-report of these experiences in children. One possible account suggests that synesthesia is present at or near birth and initially binds basic shapes and forms to colors, which are later refined to grapheme-color associations through experience. Consistent with this view, studies show that similarly shaped letters and numbers tend to elicit similar colors in synesthesia and that some synesthetes consciously associate basic shapes with colors; research additionally suggests that synesthetic colors can emerge for newly learned characters with repeated presentation. This model further predicts that the initial shape-color correspondences in synesthesia may persist as implicit associations, driving the acquisition of colors for novel characters. To examine the presence of latent color associations for novel characters, synesthetes and controls were trained on pre-defined associations between colors and complex shapes, on the assumption that the prescribed shape-color correspondences would on average differ from implicit synesthetic associations. Results revealed synesthetes were less accurate than controls to learn novel shape-color associations, consistent with our suggestion that implicit form-color associations conflicted with the learned pairings.

## Introduction

Synesthesia is an involuntary experience in which stimulation of one sensory modality causes activation in a second, separate modality. In two of the most common forms, hearing sounds or viewing graphemes (letters and numbers) elicits the experience of a specific color (sound-color and grapheme-color synesthesias; Baron-Cohen et al., 1996; Cytowic and Eagleman, 2009). For example, to synesthete EA the letter C always appears a vivid yellow, irrespective of its actual color. These sensory percepts typically begin early in childhood and remain extremely consistent throughout one's lifetime. Further, synesthesia runs in families (Baron-Cohen et al., 1996; Ward and Simner, 2005; Asher et al., 2009; Brang and Ramachandran, 2011), suggesting it is a heritable trait. While research over the past decade has done well to establish the validity of synesthetes' subjective reports (e.g., Ramachandran and Hubbard, 2001a) as well as the perceptual reality of these experiences (Palmeri et al., 2002), the manner in which synesthetic associations develop from infancy to adulthood remains an active matter of debate.

The neural substrates underlying grapheme-color synesthesia have been thoroughly studied using both psychophysical tests and functional imaging (Nunn et al., 2002; Palmeri et al., 2002; Hubbard et al., 2005). Upon viewing achromatic numbers or letters, grapheme-color synesthetes show co-activation of grapheme regions in the posterior temporal lobe and color area V4, which has been suggested to give rise to the concurrent sensation of color (Hubbard et al., 2005; Sperling et al., 2006). This pattern of activation in synesthetes is consistent with research on crossmodal integration in the normal population revealing the engagement of early sensory regions in multimodal processes (Driver and Spence, 2000; Schroeder and Foxe, 2005; Kayser and Logothetis, 2007). Ramachandran and Hubbard (2001a) proposed that this cross-activation is driven by an excess of neural connections in synesthetes, possibly due to decreases in neural pruning between typically interconnected areas, with a recent update of this model introduced by Brang et al. (2010). In support of this theory, a number of studies have demonstrated anatomical differences in the inferior temporal lobes of synesthetes, near regions related to grapheme and color processing, including increased fractional anisotropy as assessed by diffusion tensor imaging (Rouw and Scholte, 2007), and increased gray matter volume, as assessed by voxel-based morphometry (Jäncke et al., 2009; Weiss and Fink, 2009).

While the developmental onset of synesthesia is a matter of active debate, it is well established that the specific synesthetic pairings (such as a yellow C for synesthete EA) evolve over time biased by statistical properties of the environment. One strong contributing factor is the native language of a synesthete, given that some letters tend to take on the colors related to prominent linguistic elements (e.g., R is typically red and G typically green based on the initial letter of the color-word in English; Simner et al., 2005) and as more frequent exposure to a particular grapheme enhances the perceived luminance of its associated synesthetic color (i.e., more frequent letters and numbers tend to be associated with more luminant synesthetic colors; Cohen Kadosh et al., 2007; Smilek et al., 2007; Beeli et al., 2008). Furthermore, some element of imprinting from early childhood may bias grapheme-color associations, such as those from colored refrigerator magnets (Witthoft and Winawer, 2013).

Given that synesthetic associations change throughout development, it has been proposed that prior to learning a character set for a particular language, synesthetic colors are initially associated with shapes and basic forms (Maurer, 1993; Brang et al., 2010; Wagner and Dobkins, 2011). Indeed, prior to learning graphemes, infants and toddlers associate Xs with the color black and Os with white (Spector and Maurer, 2008). These shape-color associations may permeate into adulthood as more similarly shaped letters evoke more similar colors in synesthesia (Brang et al., 2011a; Jürgens and Nikolić, 2012; Watson et al., 2012) thought to occur from latent shape-color associations. In addition, some synesthetes report varying saturation of synesthetic colors by letters of different case and font suggesting an import of letter shape.

Contrary to this view, several studies highlight the influence of semantic information on synesthetic colors (e.g., Mroczko et al., 2009; Jürgens and Nikolić, 2012) and instead propose that graphemes evoke ideas which in turn elicit synesthetic colors (i.e., “ideaesthesia”). In this model, low-level elements of a grapheme, such as shape, do not evoke synesthetic colors on their own. Instead synesthetic colors are evoked only when the synesthete extracts semantic meaning from the grapheme in isolation or in the context of a word or sentence (Jürgens and Nikolić, 2012). Indeed, synesthetic colors are modulated by context, such that an ambiguous letter evokes the color corresponding to the perceived letter (e.g., Ramachandran and Hubbard, 2001b).

As synesthetic associations tend to transfer to novel learned scripts (e.g., Mroczko et al., 2009) and as synesthetes can learn new associations (Jürgens and Nikolić, 2012; Blair and Berryhill, 2013) the question remains of whether latent color associations exist for the novel scripts based on their form (such that shapes in the novel character set that are similar to letters and numbers will take on similar colors). To test for the presence of latent color associations for novel characters, 15 synesthetes and 15 controls were trained to learn novel shape-color corresponances with the goal of causing conflict with latent associations, as the proscribed shape-color correspondences would on average differ from implicit synesthetic associations.

## Materials and methods

### Participants

Data were collected from 15 synesthetes (mean age = 23.0; 13 females; 14 right-handed) and 15 non-synesthetic controls (mean age = 20.2; 10 females; 15 right-handed). All participants were fluent English speakers. Synesthetes were recruited via fliers posted on the UCSD campus, as well as similar ads on the web. Synesthesia was confirmed by means of consistency matching as well as reaction time testing for color congruency, standardized by Eagleman et al. (2007). All participants gave signed informed consent prior to the experiment, and participated either for cash or in fulfillment of course requirement. None of the control participants reported any known forms of synesthesia.

### Design and procedure

**Figure 1:**
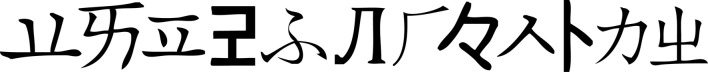
**Grapheme set from which synesthetes selected six shapes that elicited no synesthetic color, were unfamiliar to the subject, and did not evoke any strong conceptual association**.

Synesthetes first selected six novel graphemes from a set of 12 (Figure 1) that met the following requirements: (1) the shape elicited no synesthetic color, (2) the shape was unfamiliar to the subject, and (3) the shape did not evoke any strong conceptual association. After grapheme-selection, one color (red, orange, yellow, green, blue, purple) was randomly paired with each one of the six novel graphemes. Subjects were instructed to memorize six novel grapheme-color associations, one grapheme with each one of the six colors, but were not informed of the specific parings. Each control's stimulus set was matched to one synesthete's set of grapheme-color stimuli.

Subjects participated in two training tasks: a Guess-and-Check paradigm and a Color Congruency paradigm. In the Guess-and-Check task, subjects were presented with one of the six graphemes on each trial and instructed to speak aloud the associated color. Black target graphemes were presented centrally in Arial font, 6° vertical visual angle, against a gray background. Graphemes were present until response, preceded by a blank screen for 250 ms, and followed immediately after response by the same grapheme in the congruent color for 1000 ms. The first six trials reflected guesses as subjects had not been previously exposed to the associations and were excluded from analyses. Two hundred and thirty four trials (39 for each grapheme) followed these initial six trials. Accuracy was scored by an experimenter present for the duration of the study and reaction time was coded by EPrime Serial Response Box microphone.

Immediately following the first task, subjects participated in the Color Congruency paradigm. Subjects were presented with the same stimuli in either a congruent or incongruent color (50% of trials each). One incongruent color was yoked to each grapheme to balance color and stimulus probabilities. Graphemes were present on screen until response and subjects responded via button presses (EPrime Serial Response Box) to indicate whether a grapheme was congruently or incongruently colored. After response, a feedback screen (1500 ms) was provided to the subjects. Subjects progressed through 240 trials in total.

### Analysis

For the Guess-and-Check paradigm, synesthetes and controls were first compared according to the total number of errors using a paired *t*-test, matching synesthetes and controls based on learned associations. To examine whether groups differed in the length of time taken to learn shape-color associations or in the persistence of errors following reaching criterion, we conducted two additional tests. First, a learning curve and 95% confidence interval were computed for each individual subject using a state-space smoothing algorithm (Smith et al., 2004) to identify the trial on which subjects reliability performed better than chance for the remainder of the experiment (0.167 probability given the six possible colors; all default parameters were used in this analysis and the toolbox can be found at www.neurostat.mit.edu/behaviorallearning). The first trial on which this criterion was reached was compared across the groups using a paired *t*-test. In order to examine the persistence of errors after reaching criterion, a paired *t*-test was used to compare synesthete's and control's accuracy on trials following the trial on which each subject reached criterion. In the color congruency paradigm, synesthetes' and controls' mean accuracy and mean response times were each compared using paired *t*-tests.

## Results

**Figure 2:**
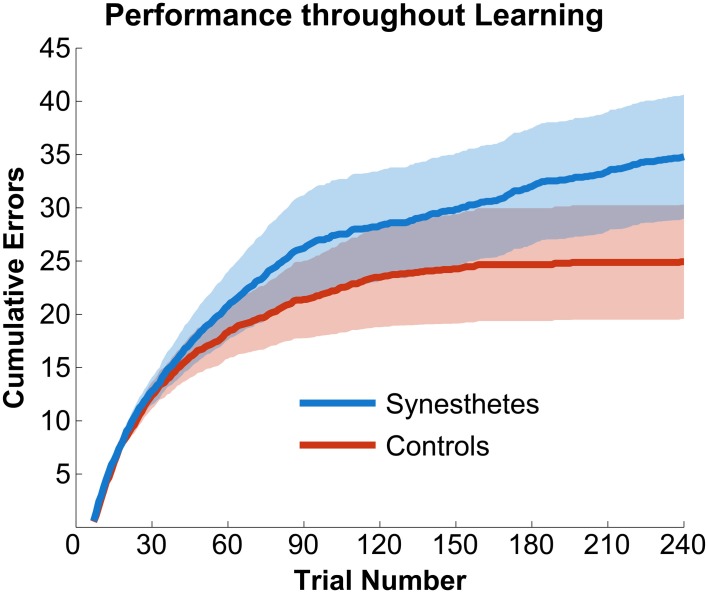
**Synesthetes (blue) and controls (red) performance as a function of time.** Solid colors reflect accuracy at each trial and partially transparent colors reflect standard errors of the mean. Of note, synesthetes continued to make errors long after controls reached a plateau. The plot begins at trial number seven as the first six trials were guesses and were excluded from all analyses.

**Figure 3:**
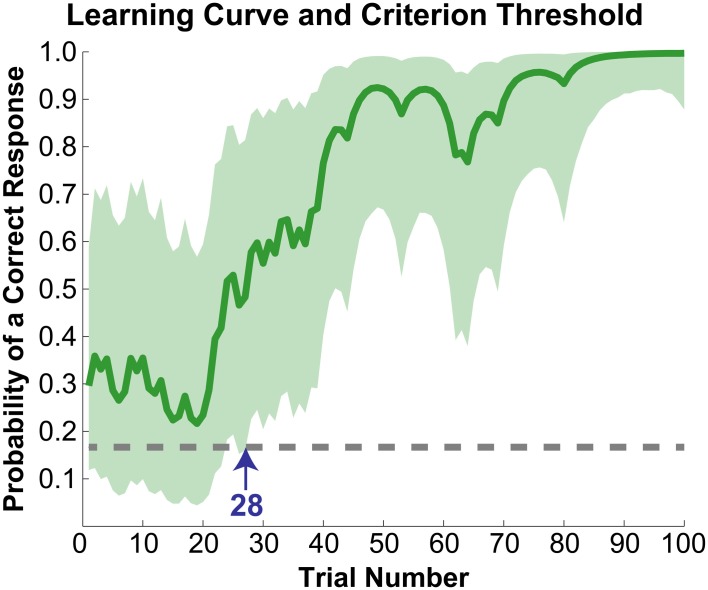
**Learning curve for a representative subject.** Solid line (dark green) reflects the probability of a correct response on any given trial. The light green cloud reflects the 95% confidence interval for this probability. Learning criterion was reached when a subject's accuracy exceeded chance (dotted gray line) with 95% certainty (marked with the blue arrow at trial number 28). The plot begins at trial number seven as the first six trials were guesses and were excluded from all analyses, and only shows results for trials up to 100 for illustrative purposes.

**Figure 4:**
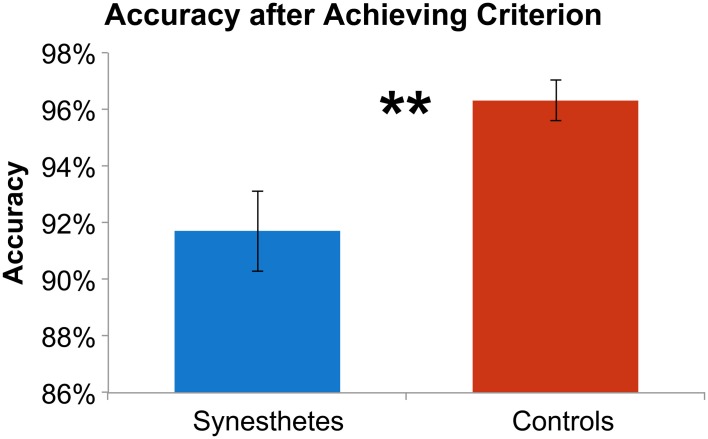
**Synesthetes (blue) and controls (red) mean accuracy for trials following reaching criterion.** Error bars reflect standard error of the mean and ^**^ denotes significance at *p* < 0.01.

The initial paradigm required subjects to learn the associated color for six novel graphemes through a guess-and-check task, with feedback. Synesthete and control groups average performance throughout the Guess-and-Check paradigm are displayed in Figure 2; also see Figure S1. In terms of the total number of mistakes made throughout this task, synesthetes exhibited a trend of a greater number of errors (mean 34.8) relative to controls (mean 24.9), *t*_(14)_ = 1.77, *p* = 0.098. In order to examine whether this difference was due to synesthetes' difficulty in the acquisition or maintenance of the novel associations over time we conducted two additional analyses. First, criterion-learning thresholds were estimated by calculating the probability of a correct response on any given trial, based on a state-space smoothing algorithm (Smith et al., 2004). This analysis identified the trial for which all subsequent responses exceeded chance performance (Figure 3), and comparison between the groups suggested no difference in learning rate between synesthetes (mean trials to criterion 24.7) and controls (mean trials 29.8), *t*_(14)_ = 0.72, *p* = 0.486. In order to examine whether synesthetes and controls differed in their ability to maintain these associations throughout the task after reaching criterion, accuracy was compared for all trials following the criterion trial. Results identified significantly impaired performance in synesthetes (mean accuracy 91.7%) relative to controls (96.3%), *t*_(14)_ = 3.59, *p* = 0.003, indicating a difficulty in maintaining these novel grapheme-color associations for synesthetes (Figure 4).

The second paradigm measured subjects' ability to identify congruently colored graphemes. Synesthetes displayed a non-significant trend of lower accuracy through this task (mean 95.6% correct) relative to controls (mean 97.8%), *t*_(14)_ = 1.65, *p* = 0.122. No reliable difference in response time was observed between synesthetes (mean 834 ms) and controls (806 ms), *t*_(14)_ = 0.42, *p* = 0.679.

## Discussion

The present paradigm examined the ability of synesthetes and controls to learn novel grapheme-color correspondences. Findings across two paradigms with the same subjects indicate that synesthetes were impaired in their ability to maintain novel, enforced grapheme-color associations. These results suggest that synesthetes are not simply better at maintaining arbitrary associations *per se*, as has been suggested previously (e.g., Ramachandran and Hubbard, 2001b), but instead argues for the presence of latent grapheme-color associations for novel character sets.

Described as the neonatal synesthesia hypotheses, one theory suggests that synesthesia is present in all individuals at or near birth and fades over time due to excessive neural pruning and re-weighting of synaptic connections (Maurer, 1993). However, as letters and number acquisition occurs relatively late in development (on average at 3 years of age; e.g., Wynn, 1990), if synesthetic associations are present early in life they most likely involve shape-color associations. In line with this, young children show consistent associations between shapes and colors (Spector and Maurer, 2008; Wagner and Dobkins, 2011) and synesthetic colors remain fluid (some letters-color associations show large variability over time) at least until age 10 (Simner et al., 2009). Indeed, these shape-based associations may permeate to synesthetic associations throughout development, as in adult synesthetes similarly shaped letters and numbers tend to have similar synesthetic colors (Brang et al., 2011a; Watson et al., 2012). Building on this evidence, Brang et al. (2010) suggested that synesthetic associations between shapes and colors remain a vestigial relic in adult synesthetes. Indeed, while it is relatively uncommon for synesthetes to show conscious shape-color associations as adults, synesthetic colors are evoked in the temporal hierarchy prior to grapheme processing as revealed by MEG (Brang et al., 2010).

As the particular associations in the present study were assigned randomly, the majority of shape-color correspondences should be in disagreement with any given synesthete's latent and unconscious association. Indeed, one synesthete admitted that for one of the novel graphemes, a conscious synesthetic color emerged partway through the task and that this color differed from the association he was attempting to learn^1^. While speculative at present, we elaborate on the neonatal synesthesia hypothesis and suggest that synesthetic associations are present early in infancy as basic form-color associations that are tuned and biased over time into grapheme-color associations, eventually losing the initial conscious pairings between colors and shapes. Nevertheless, while these results suggest a latent influence of shapes on synesthetic colors, research demonstrates that colors are modulated by numerous factors including context (Ramachandran and Hubbard, 2001b; Dixon et al., 2006; Brang et al., 2008, 2011b), phonemic similarity (Asano and Yokosawa, 2012) and environment (Witthoft and Winawer, 2013).

Past studies in support of the “ideaesthesia” view of synesthesia, that synesthetic colors are evoked by semantic concepts as opposed to low-level stimulus properties, demonstrate that an arbitrary synesthetic color is able to transfer to a novel grapheme through semantic knowledge (Mroczko et al., 2009) and suggest that novel graphemes can take on any arbitrary color. Consistent with this finding, here synesthetes successfully learned arbitrary shape-color correspondences. Critically, however, this process was significantly more difficult in synesthetes compared to controls, suggesting latent color preferences in synesthetes conflicted with the enforced associations.

Grapheme-color synesthetes demonstrate enhanced memory recall for word lists that evoke synesthetic colors, line drawings that do not evoke synesthetic colors, and for memory of colors themselves (Yaro and Ward, 2007; Rothen and Meier, 2010; Gibson et al., 2012). However, memory enhancements in synesthesia are generally restricted to colors and visual stimuli and are not a generalized trait (for a review see Rothen et al., 2012). The present results further argue against a generalized memory enhancement in grapheme-color synesthetes suggesting they are not simply better at making arbitrary associations between complex shapes and colors. In addition, one possible explanation of why synesthetic colors transfer to novel symbols according to shape similarity is that this serves as a memory mnemonic, in that the color provides an extra cue leading to the remembrance of the shape of the grapheme. However, in these results synesthetes demonstrated impaired acquisition of the associations which would have utilized this approach.

One shortcoming of the present study is the absence of a within-group control demonstrating the specificity of this finding to confirm that grapheme-color synesthetes are not generally impaired in making novel associations. While these data cannot exclude such a possibility, future research may be able to rule out this possibility by examining synesthetes' ability to make color-color or shape-shape associations (in which we would expect no difference between synesthetes and controls). However, as mentioned above, synesthetes demonstrate better color memory in general (Yaro and Ward, 2007), which may instead facilitate synesthetes' ability to make color-color associations.

In sum, the current results suggest that synesthetic colors are biased in accordance with latent shape-color associations, and is consistent with views of this phenomenon emerging early in infancy with colors initially being evoked by shapes and basic forms. Future studies should seek to predict novel grapheme-color correspondences based on a synesthete's present set of number and letter-color associations to more directly bias subjects' ability to acquire these novel pairings. Given research suggesting a benefit of synesthetic colors on memory we expect that these novel associations will be better maintained if they are paired with synesthetic colors in line with those of their latent shape-color associations.

## Conflict of interest statement

The authors declare that the research was conducted in the absence of any commercial or financial relationships that could be construed as a potential conflict of interest.

## Footnotes

^1^Unfortunately we did not systematically ask our synesthetes whether this was a common phenomenon, necessitating additional research on this topic.

## Supplementary material

Figure S1 **Individual subject plots showing synesthetes' (blue) and controls' (red) data points for each of the three analyses conducted for the Guess-and-Check paradigm (A–C) and for the Congruency paradigm.** (D) Subject numbers are labeled along the x-axis. **^**^*p* < 0.01.**
