# Supplementary material for: Impaired acquisition of novel grapheme-color correspondences in synesthesia
Source: Front Hum Neurosci. 2013 Oct 30;7:717. doi: 10.3389/fnhum.2013.00717 (PMC3812534; doi:10.3389/fnhum.2013.00717)
Supplement: Figure S1 — Individual subject plots showing synesthetes' (blue) and controls' (red) data points for each of the three analyses conducted for the Guess-and-Check paradigm (A–C) and for the Congruency paradigm. (D) Subject numbers are labeled along the x-axis. **p < 0.01. [file Presentation1.PDF]

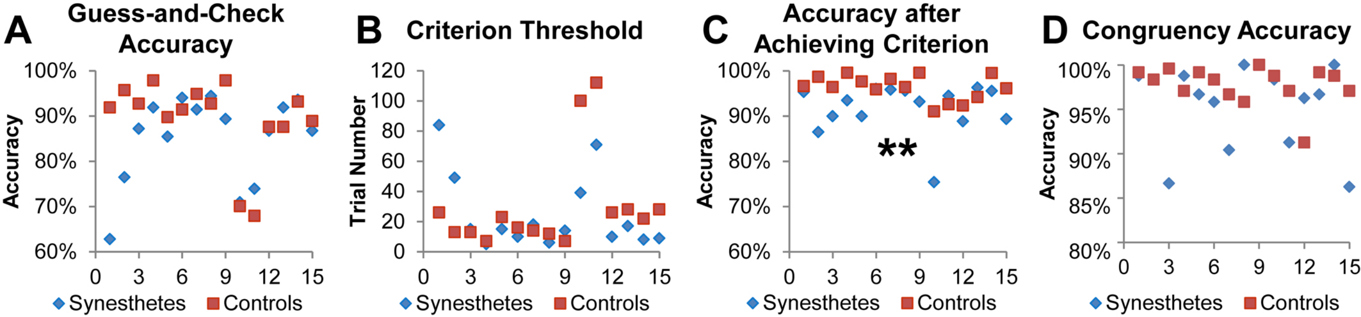

A
Guess-and-Check Accuracy
Accuracy
100%
90%
80%
70%
60%
0
3
6
9
12
15
Synesthetes
Controls
B
Criterion Threshold
Trial Number
120
100
80
60
40
20
0
0
3
6
9
12
15
Synesthetes
Controls
C
Accuracy after Achieving Criterion
Accuracy
100%
90%
80%
70%
60%
**
0
3
6
9
12
15
Synesthetes
Controls
D
Congruency Accuracy
Accuracy
100%
95%
90%
85%
80%
0
3
6
9
12
15
Synesthetes
Controls
